# Supplementary figures and images for: Measuring S-Phase Duration from Asynchronous Cells Using Dual EdU-BrdU Pulse-Chase Labeling Flow Cytometry
Source: Genes (Basel). 2022 Feb 24;13(3):408. doi: 10.3390/genes13030408 (PMC8951228; doi:10.3390/genes13030408)

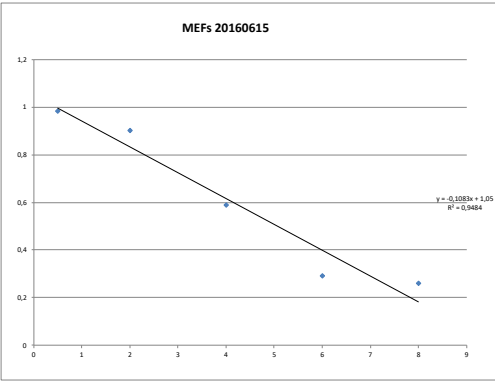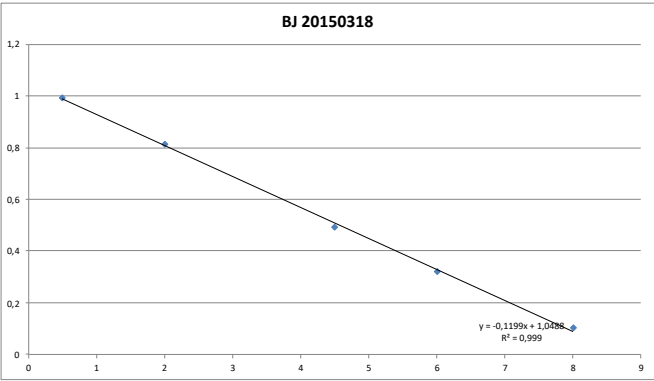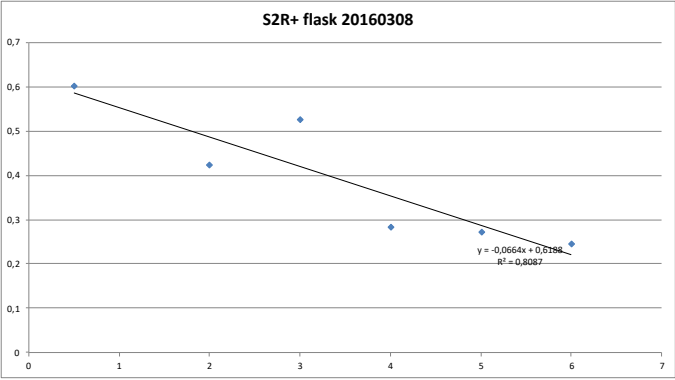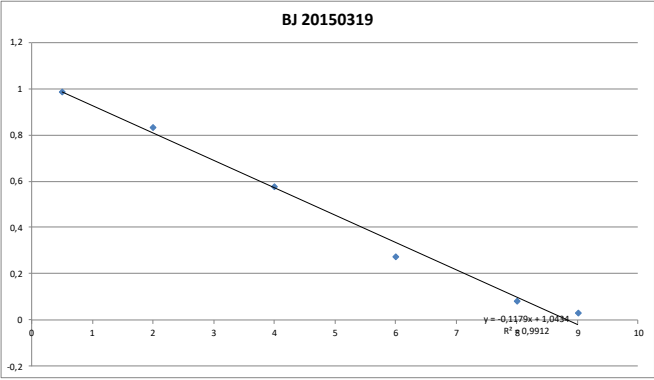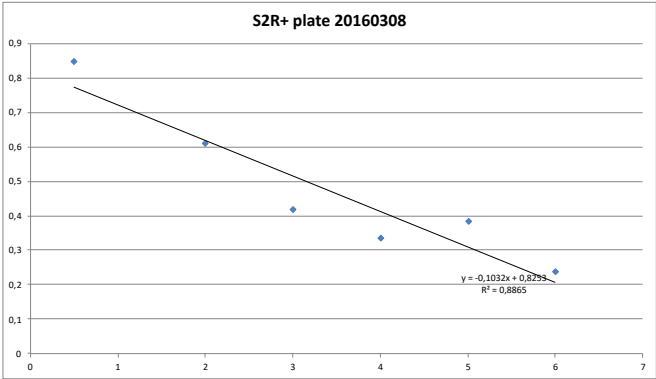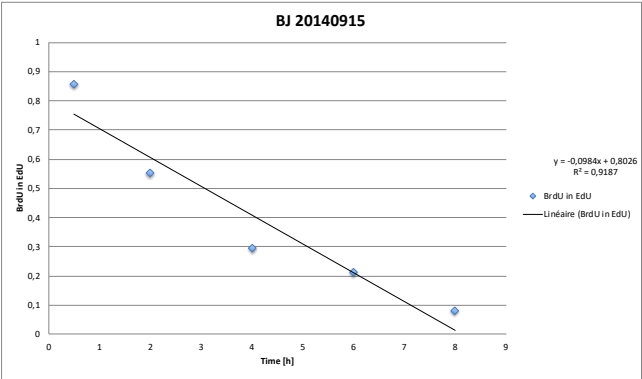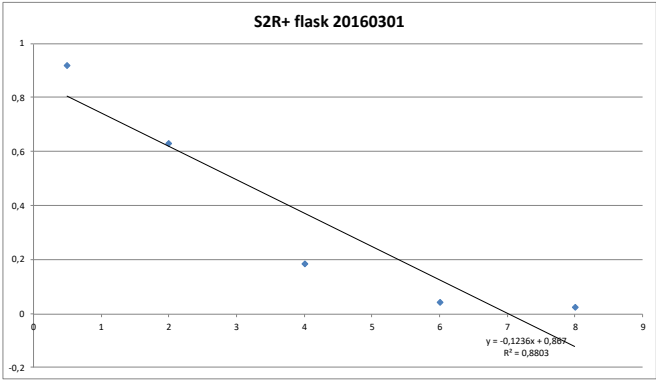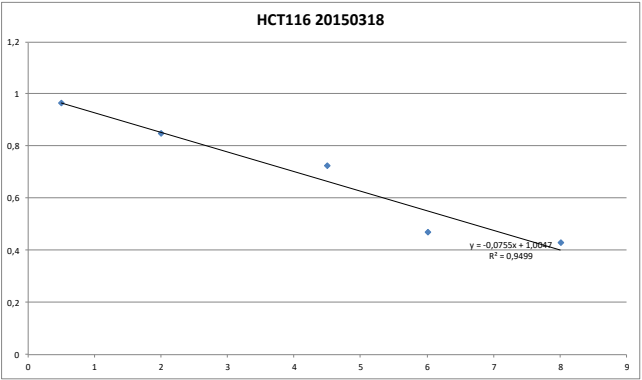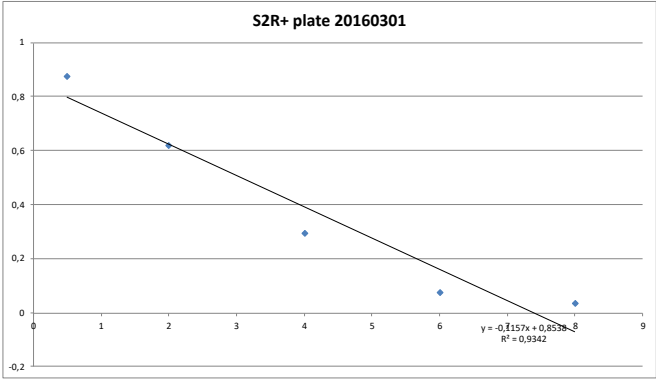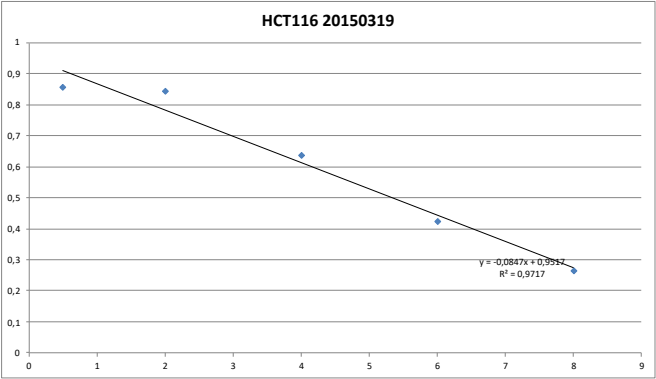

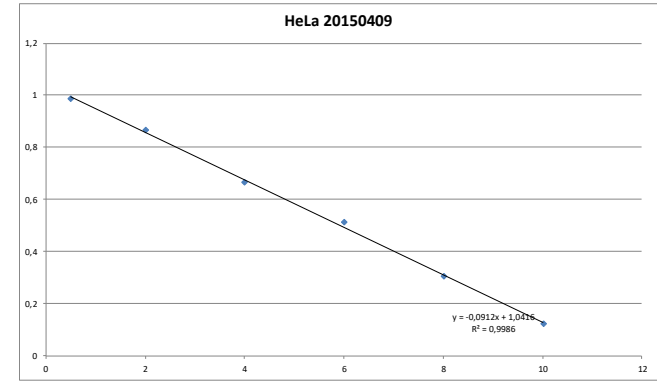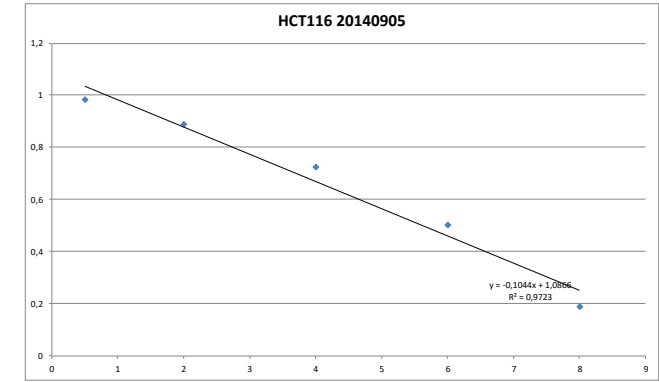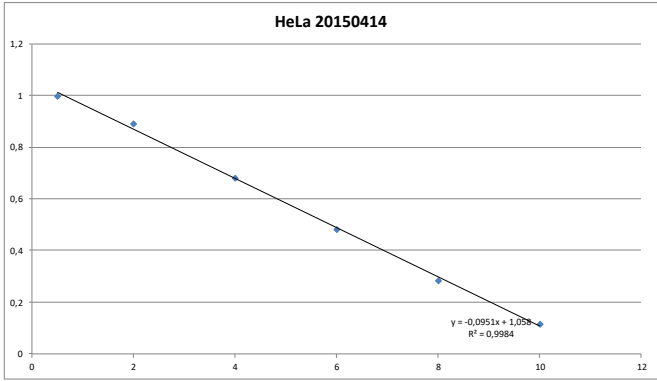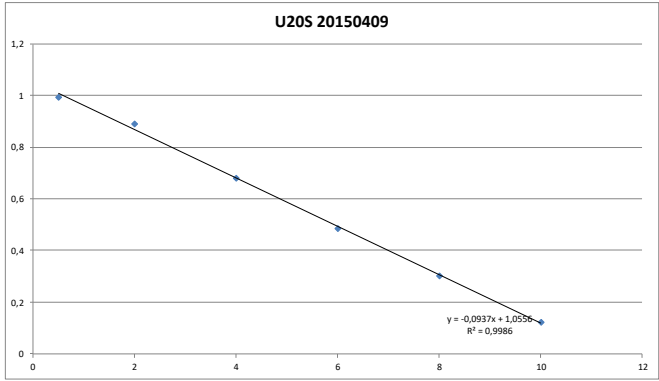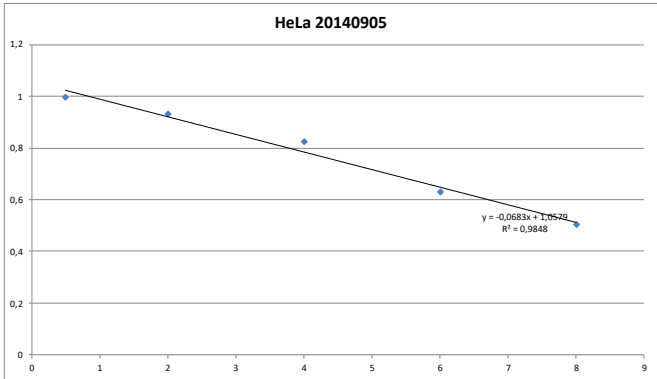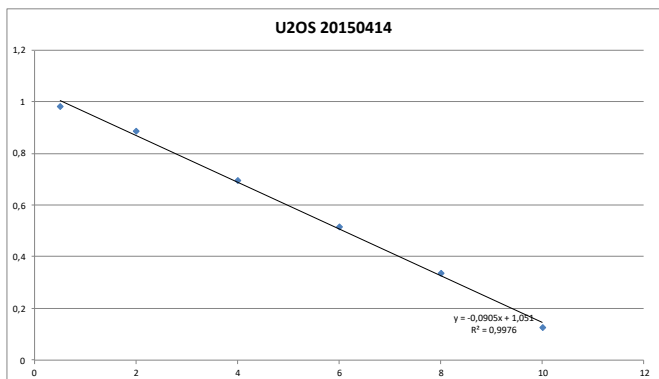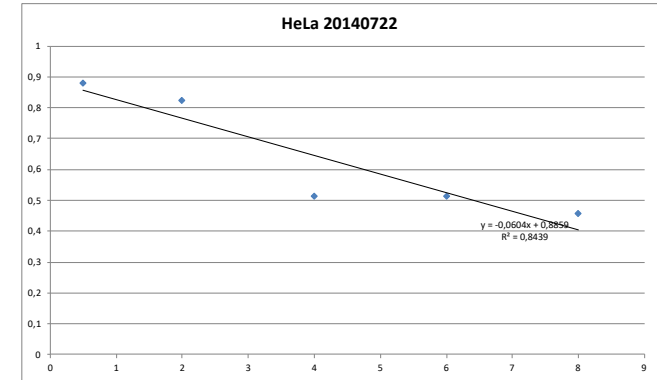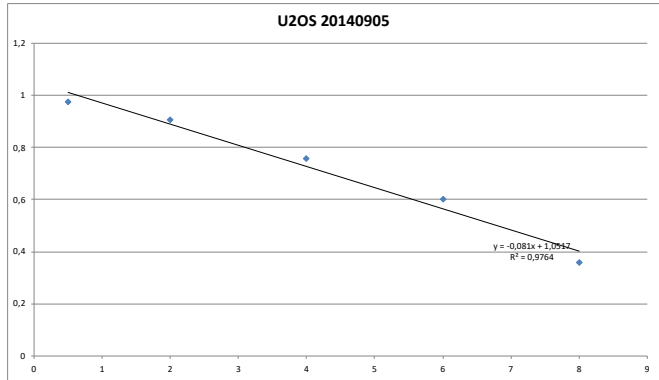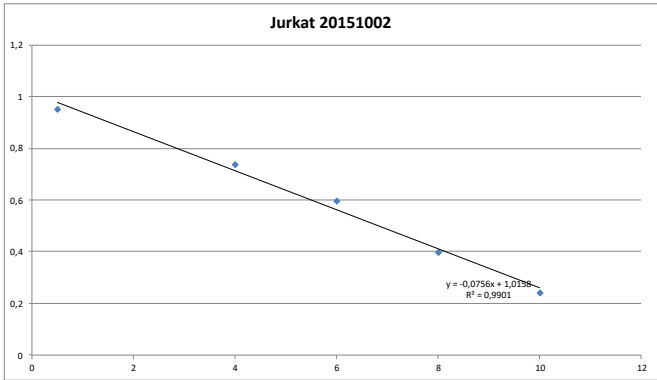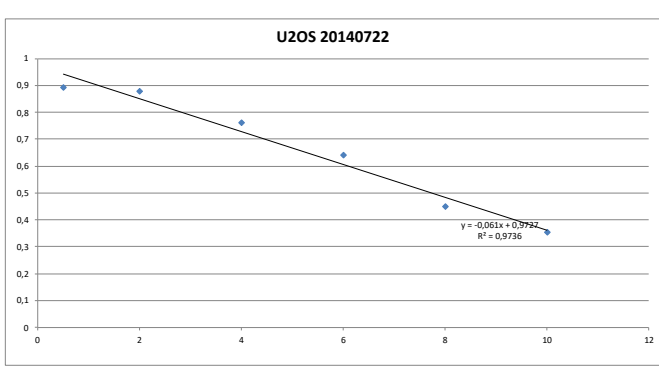

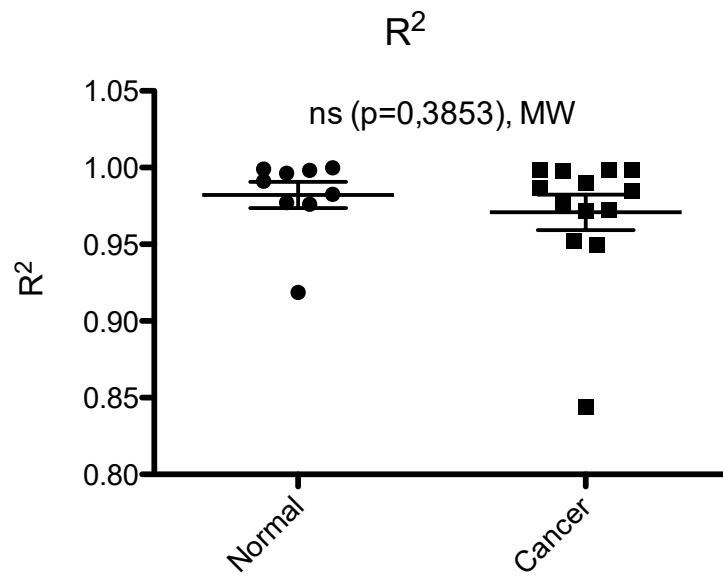

Bialic et al., SuppFigure S1

Supplement: Supplementary file 1 [file genes-13-00408-s001.zip › genes-1579367-supplementary.pdf]
